# Acceptability of digital health interventions: embracing the complexity

**DOI:** 10.1093/tbm/ibab048

**Published:** 2021-05-08

**Authors:** Olga Perski, Camille E Short

**Affiliations:** 1 Department of Behavioural Science and Health, University College London, London, UK; 2 Melbourne Centre for Behaviour Change, Melbourne School of Psychological Sciences, University of Melbourne, Melbourne, Australia; 3 Melbourne School of Health Sciences, University of Melbourne, Melbourne, Australia

**Keywords:** Acceptability, Complexity, eHealth, Engagement, mHealth, Technology acceptance

## Abstract

Acceptability is a core concept in digital health. Available frameworks have not clearly articulated *why* and *how* researchers, practitioners and policy makers may wish to study the concept of acceptability. Here, we aim to discuss (i) the ways in which acceptability might differ from closely related concepts, including user engagement; (ii) the utility of the concept of acceptability in digital health research and practice; (iii) social and cultural norms that influence acceptability; and (iv) pragmatic means of measuring acceptability, within and beyond the research process. Our intention is not to offer solutions to these open questions but to initiate a debate within the digital health community. We conducted a narrative review of theoretical and empirical examples from the literature. First, we argue that acceptability may usefully be considered an emergent property of a complex, adaptive system of interacting components (e.g., affective attitude, beliefs), which in turn influences (and is influenced by) user engagement. Second, acceptability is important due to its ability to predict and explain key outcomes of interest, including user engagement and intervention effectiveness. Third, precisely what people find acceptable is deeply contextualized and interlinked with prevailing social and cultural norms. Understanding and designing for such norms (e.g., through drawing on principles of user centered design) is therefore key. Finally, there is a lack of standard acceptability measures and thresholds. Star ratings coupled with free-text responses may provide a pragmatic means of capturing acceptability. Acceptability is a multifaceted concept, which may usefully be studied with a complexity science lens.

## Teaser

Acceptability of digital health interventions is a multifaceted concept, which may usefully be studied with a complexity science lens.

**Implications**

**Practice:** Care should be taken to distinguish between acceptability of trial procedures (e.g., randomization, follow-up assessments) and of the intervention itself.

**Policy:** The utility of the concept of acceptability lies in its ability to predict and explain key outcomes of interest, such as user engagement, intervention effectiveness and widespread adoption.

**Research:** The suggested dynamic model is intended to serve as a starting point for empirical research examining the relationship between the closely related concepts of acceptability, user engagement and intervention effectiveness.

## BACKGROUND

The extent to which interventions are perceived as “acceptable” to patients, family members, treatment providers, institutional review boards and policy makers is central to digital health research and practice [1]. Acceptability sits at the core of the widely deployed Technology Acceptance Model [2], which posits that perceived ease of use and perceived usefulness of a given technology positively influence usage intentions, which in turn drive the adoption of new technologies. Acceptability is related to the term “tolerability” in the medical setting, which has historically been used to indicate the extent to which a drug or procedure induces pain, discomfort, side effects or adverse events [3]. However, acceptability and tolerability are not considered identical. A plethora of definitions and measures of acceptability are available in the digital health literature [4], with extant frameworks and theorizations converging on the view that acceptability primarily captures how people think and feel about a given digital health intervention [4, 5]. Although available frameworks provide useful overviews of how acceptability has been defined and measured, it is our view that they have not clearly articulated *why* and *how* researchers, practitioners and policy makers may wish to study the concept of acceptability. In addition, a discussion of the wider social and cultural norms that influence whether or not a given technology is perceived as acceptable is lacking. Therefore, we aim to discuss (i) the ways in which acceptability might differ from closely related concepts, such as user engagement; (ii) the utility of the concept of acceptability in digital health research and practice; (iii) social and cultural norms that influence acceptability; and (iv) pragmatic means of measuring acceptability, within and beyond the research process. Our intention is not to offer solutions to these open questions but to initiate a debate within the digital health community.

### The ways in which acceptability might differ from closely related concepts, such as user engagement

**Fig 1:**
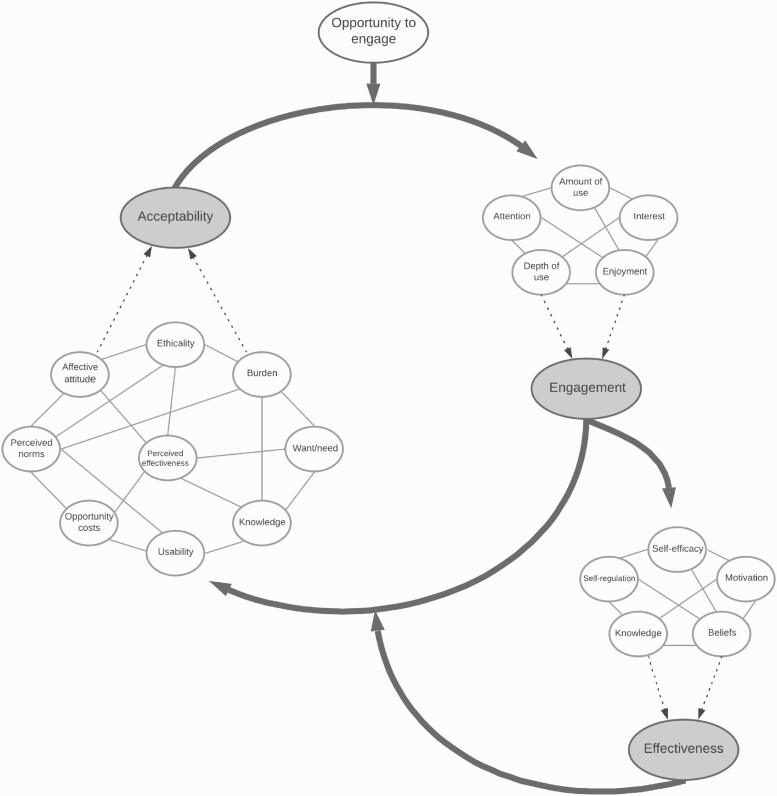
Suggested dynamic model linking the concepts of acceptability (an emergent property of a complex, adaptive system of interacting components), user engagement and intervention effectiveness. Inspired by the theoretical and conceptual frameworks by Sekhon et al. [5], and Perski et al. [6], in addition to the graphical representation of interaction-dominant systems by Hilpert and Marchand [7]. The transparent ovals represent a non-exhaustive network (a set of “best bets”) of interacting components. The dotted arrows leading to the grey ovals represent higher-level, emergent properties, arising from the interaction of components at a lower level. The thick, solid arrows indicate influences.

There is no consensus definition of acceptability. In the literature, acceptability has been defined as people’s affective attitudes toward a new digital health intervention, usage intentions (e.g., willingness to engage with the intervention), actual usage (e.g., frequent interaction with the intervention), and satisfaction after having engaged with the intervention [4]. It has also been noted that perceptions of acceptability can be formed (i) after learning about a new intervention but before having engaged with it (referred to as “pre-use acceptability” [4] or “prospective acceptability” [5]), (ii) during engagement with the intervention (“initial use acceptance” [4] or “concurrent acceptability” [5]) and (iii) after a period of engagement (“sustained use acceptance” [4] or “retrospective acceptability” [5]). The expression of acceptability as a dynamic process raises the question as to *what form* acceptability takes—for example, whether it is usefully expressed as an attitude, a belief, a behavior, or all of the above—and how it differs from closely related concepts, such as user engagement and satisfaction. Although there is also no consensus definition of user engagement, it has been proposed that it is a multidimensional construct with behavioral (e.g., the frequency, amount, depth and duration of intervention use), cognitive and affective facets (e.g., attention, interest, and enjoyment while interacting with the intervention) [6]. Similarly, acceptability is thought to reflect how people think and feel about the technology before, during and after having engaged with it [4, 5]. We therefore propose that acceptability may usefully be considered an emergent property of a complex, adaptive system of interacting components (e.g., beliefs, knowledge, affective attitude), which in turn influences (and is influenced by) user engagement and intervention effectiveness (see Fig. 1).

Before outlining the suggested dynamic model linking acceptability, engagement and effectiveness, we need to take a brief detour to complexity science. Complex, adaptive systems are characterized by being made up of multiple, interacting parts (i.e., “interaction-dominant” systems) and tend to display higher-order, emergent properties, such as a sudden change in behavior or insight, which arise from interactions at a lower level of the system [7–9]. Such emergent properties are greater than the sum of their parts and are hence not reducible to the individual system components. We argue that acceptability may usefully be considered an emergent property of a complex, adaptive system of knowledge, beliefs and attitudes, experienced by the individual as a gut reaction or sudden insight (e.g., “I like the sound of that” or “No, thanks!”) [8, 9]. Within our suggested dynamic model, upon learning about a new digital health intervention (e.g., a smartphone app, an implant, a wearable device), potential users consider whether the intervention fits with their value system (“ethicality”), whether it appears effortful to use (“burden”) and/or whether it appears likely to achieve its purpose (“perceived effectiveness”) [5]. Such beliefs are themselves heavily influenced by the sociocultural context within which the individual resides (“perceived norms”). Potential users simultaneously form an impression (“affective attitude”) of how they feel about the intervention, which is positively or negatively valanced, which influences (and is influenced by) their motivation to change (“want/need”). The interaction of these components – cognitions and affect – gives rise to pre-use acceptability (i.e. an emergent property of the complex, adaptive system), which is experienced as a gut reaction [8, 9]. Individuals then need to have the opportunity to engage with the intervention—the digital health intervention may yet need to be developed. During or immediately following engagement with the intervention (which itself can be considered an emergent property of a complex, adaptive system), cognitions and affect are updated, which may in turn lead to increased/reduced acceptability (or no change), increased/reduced motivation to use (or no change) or increased/reduced engagement (or no change). In line with available frameworks, engagement is thought to be linked to intervention effectiveness through exposure to the intervention’s active ingredients [6].

The suggested dynamic model is intended to serve as a starting point for empirical research examining the relationship between the closely related concepts of acceptability, user engagement and intervention effectiveness. The move from a conceptual to a mechanistic (or statistical) model may require the application of dynamic systems modelling [7, 10]. Although the proposed dynamic model has been described here in relation to potential or actual users, it is also intended to apply to family members, caregivers and healthcare professionals.

### The utility of acceptability in digital health research and practice

From a public health (as opposed to, for example, a philosophical) perspective, it can be argued that the utility of the concept of intervention acceptability lies in its ability to predict and explain key outcomes of interest, such as user engagement, intervention effectiveness and widespread adoption at the local, national and international level. At the individual level, we know that people need to engage with digital health interventions for them to be effective [11]; however, engagement tends to be suboptimal [11–13]. This low engagement may, at least partly, be related to low acceptability. At the population level, the failure to implement evidence-based digital health interventions within healthcare or organizational systems is widespread [14]. This early abandonment of digital health interventions, which have typically been developed and evaluated with some degree of government funding, is wasteful. Extensive research with qualitative and quantitative data collected at the micro- (i.e., individual technology users), meso- (i.e., organizational systems), and macrolevel (i.e., national or international policy) shows that a complex web of factors contribute to the (un-)successful adoption of digital health interventions [14], with acceptability touted as a necessary (but not sufficient) condition for success. The role of acceptability within health and social care is also reflected in the UK Medical Research Council’s guidance for the development and evaluation of complex health interventions [15]: pilot and feasibility studies, which typically aim to evaluate intervention acceptability and feasibility of recruitment, are considered a standard requirement before moving to more lengthy and costly randomized controlled trials (RCTs) and implementation/ecological trials [16, 17].

It should, however, be noted that intervention acceptability may sometimes be conflated with the acceptability of trial procedures (e.g., randomization, completion of frequent follow-up assessments). For example, a common reason for patients to decline clinical trial participation (which tends to be interpreted as a sign of low acceptability) is because they do not wish to undergo randomization [18]. A core reason why randomization may not be perceived as acceptable in those who decline trial participation is because of perceptions of clinical superiority (as opposed to equipoise), with the new intervention assumed to be better than treatment as usual [18]. Care should therefore be taken to distinguish between acceptability of the trial procedures and of the intervention itself, as the user may find a given digital health intervention acceptable but not be willing to undergo randomization or complete frequent follow-up assessments. It would therefore be useful for researchers to capture how frequently potential participants decline to enroll in, or drop out from, research studies involving new digital health interventions due to low intervention acceptability (as opposed to low acceptability of trial procedures). Interviews or brief surveys with participants who decided to enroll and those who decided against taking part (or dropped out) could help disentangle this [19, 20]. For example, reasons for declining participation in an RCT of an exergame for older adults were collected with free-text responses and coded into ten categories (e.g., unwilling to attend follow-up assessments, unwilling to use the exergame device) [21]. However, as attempts to contact participants who declined to participate at a future time point may be unsuccessful, it is recommended that researchers try to capture reasons for/against participation as close in time as possible to when the decision was made.

This consideration notwithstanding, it is arguably important to examine whether end-users (e.g., patients, family members, clinicians) perceive a digital health intervention as acceptable due to its likely influence on key public health outcomes, including engagement, effectiveness and the scaling up of new technologies.

### Social and cultural norms that influence acceptability

We further argue that the specific intervention components or design elements that are perceived as acceptable are likely to change over time and across contexts due to changing social and cultural norms, which may themselves differ depending on socioeconomic position, ethnicity or geographic region [22]. For example, new technologies such as chatbots (i.e., conversational agents underpinned by more or less sophisticated machine learning algorithms) and wearables (i.e., devices worn by users with a view to capturing real-time information about health and wellbeing) are frequently introduced on the market and used as part of digital health interventions. Research shows that although chatbots were seen as moderately acceptable by internet users in 2019, there was hesitancy regarding their information quality, accuracy and trustworthiness [23]. Similar trends have been observed for wearable devices and artificial intelligence (AI) in healthcare [24]. Due to evolving social and cultural norms, however, chatbots underpinned by AI may at present be seen as “uncanny” by the majority of users [25], but commonplace and more widely acceptable in 2030. In addition, research shows that users tend to rely heavily on “social proof,” including other users’ ratings or recommendations from healthcare professionals, to help navigate which digital health interventions to adopt [26, 27], as these serve as shortcuts for selecting interventions that others (who are presumably similar to oneself) perceive as acceptable.

As acceptability is influenced by prevailing social and cultural norms, which are deeply contextual and ever changing, it has been argued that those who want to accelerate the adoption of new technologies within their organization should invest in “early adopters” (i.e., trendsetters who differ from the majority in that they are not dependent on social proofing) and make their activities observable in an attempt to change social and cultural norms [28]. Torous et al. [29] have introduced the notion of “digital navigators,” a dedicated job role within mental health services to ensure patients and carers are introduced to new digital health interventions by a trusted individual and are able to comfortably use new technologies, with a view to increasing their adoption. However, as is standard practice in digital health, understanding and designing for prevailing social and cultural norms (e.g., circumventing or attempting to alter these) also needs to be addressed at the early stages of the design process.

Principles of user centered and participatory design are critical for understanding potential users’ prevailing norms, values and goals, and ensuring that acceptable interventions are designed from the outset. Rather than the user being a passive subject of study, with the researcher bringing knowledge from theory and gathering additional information about user needs through observation and interviews, it is important that the people who will go on to use the intervention play a key role in the ideas generation and concept development [30]. For example, Easton and colleagues co-created an autonomous virtual agent for and with people living with chronic health conditions through a series of co-design workshops [31]. Activities such as persona worksheets and “a day in the life” exercises were used to identify design concepts that were acceptable to the target users. However, Papoutsi et al. also raise issues of “mainstreaming” [32], with co-design activities carried out only within a specific clinic or user group potentially leading to issues of low acceptability when the intervention is to be used in a new setting.

### Pragmatic means of measuring acceptability, within and beyond the research process

There is a lack of validated measures and thresholds against which to determine whether a digital health intervention is perceived as acceptable by users [4]. This is problematic – when deciding whether or not to progress to a large-scale RCT after the completion of a pilot or feasibility study, researchers and practitioners often use common sense or set their own criteria for determining whether the intervention was considered acceptable. An informal search for available acceptability measures and pilot or feasibility studies of digital health interventions conducted between 2015 and 2020 showed that none of the available acceptability measures had a theoretically or empirically established cut-off [33–37]. In addition, only one [38] out of 14 identified pilot or feasibility studies used an a priori cut-off to determine whether the intervention was considered acceptable [39–51]. This apparent lack of cut-offs notwithstanding, 14/14 of the identified pilot or feasibility studies concluded that the intervention was moderately to highly acceptable. We also note that acceptability is often conflated with usability or satisfaction in digital health research, with usability scales often being deployed to capture intervention acceptability in empirical research [4, 52]. With acceptability considered an emergent property of a complex, adaptive system, it is important to identify pragmatic ways of measuring it within and beyond the research setting. Although it may be possible to develop and validate an acceptability questionnaire, it is currently unclear how such a questionnaire would get at the construct of interest (i.e., acceptability as an emergent property), as opposed to the factors that may interact to give rise to the construct (e.g., usability, perceived effectiveness, ethicality). Instead, a brief star rating (similar to those used in commercial app stores) with an accompanying free-text response, think aloud methodology, interviews or focus groups may usefully capture the acceptability of digital health interventions.

First, to illustrate the potential utility of star ratings with accompanying free-text responses, we conducted an informal analysis of the first five app reviews that appeared alongside star ratings in the Apple App Store and Google Play Store for i) the first top rated physical activity and stop smoking apps and ii) the first physical activity and stop smoking apps with an average rating of three stars or less (see Table 1). The methodology was selected to capture a variety of ratings and reviews. Low star ratings (i.e., 1–3) tended to be accompanied by reports of usability issues (e.g., bugs, crashes, failure to sync with wearable devices) and low perceived effectiveness, which evoked strong negative reactions in the users (e.g., “nothing drives me crazier,” “annoyed”). High ratings (i.e., 4–5) tended to be accompanied by expressions of perceived effectiveness of the app, with few or no usability issues reported, and expressions of positive reactions (e.g., “crazy this is free!”).

**Table 1:** Example reviews for top and mid-rated physical activity and smoking cessation apps across the Apple App Store and Google Play Store

	Physical activity		Smoking cessation	
	Top rated	Three stars or less	Top rated	Three stars or less
Apple App Store	“Very helpful.” ^a^	“Crashes when trying to open app every time.” ^c^	“Really helped me quit as it was great to see my progression as the weeks rolled by.” ^a^	“Annoyed since the update has deleted my previous time 2+ years without smoking. Cheers guys.” ^c^
	“Crazy this is free! So good.” ^a,b^	“App keeps crashing. Same for years.” ^c^	“…the best part is it’s so easy to navigate!” ^c^	“The calculator is wrong.” ^a,c^
Google Play Store	“Reliably works. I like that the screen briefly shows the time of day.” ^c^	“…nothing drives me crazier than poorly built applications.” ^c^	“Really helpful, gives excellent info, advice and constant support.” ^a^	“How does it work?” ^c^
	“Works very well but make sure you don’t have power saving mode on.” ^c^	“They need to fix the distance calculation. It seems I get a mile and a half for every mile I walk.” ^a,c^	“This app helped me so much. I smoked for ten years and now 3 months smoke free.” ^a^	“Unable to add photo from gallery.” ^c^

^a^ Perceived effectiveness.

^b^ Opportunity costs.

^c^ Usability.

**Fig 2:**
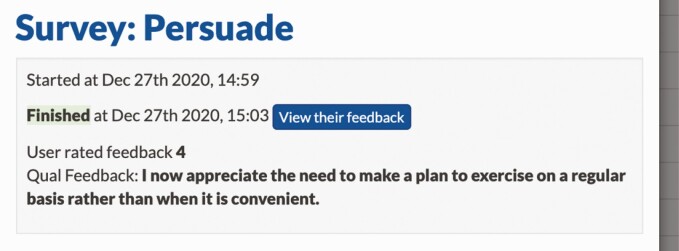
Example rating and free-text comment in ExerciseGuide [53].

In addition, simple ratings paired with free-text responses have recently been deployed in the research setting. In an ongoing pilot RCT of ExerciseGuide [53], a web- and telephone-based physical activity intervention for people living with metastatic prostate cancer, acceptability of the different intervention components is examined with a 5-point rating which is elaborated on in a free-text comment (see Fig. 2). This type of information can also be gleaned from user centered design activities to understand either pre-use (e.g., during early design workshops) and concurrent acceptability (e.g., during user testing sessions when asking for feedback on early prototypes).

Further work is needed to establish whether the use of brief star ratings and accompanying free-text responses provides a sufficiently precise measure of acceptability, and whether an acceptability cut-off (e.g., four out of five stars) could be established. However, they appear to have initial face validity in that they provide useful information about people’s reactions to digital health interventions and key factors that influence these. It would also be important to examine whether average ratings across users, and for particular subgroups of users, predict user engagement or acceptability in new users. Whether or not findings generalize to new users in different contexts is an empirical question, which merits further investigation. For example, one may predict that an app with high star ratings and few usability issues reported will evoke positive reactions in new users. However, if relying on star ratings and reviews on commercial app stores (as opposed to collecting data as part of a research project), researchers may first need to identify and filter out staged (or fake) reviews and be wary that the overall distribution of reviews is likely bimodal, with a bias toward highly positive and negative reviews [54].

Second, qualitative methods including think aloud methodology, interviews or focus groups may usefully complement quantitative acceptability ratings. For example, interviews and think aloud methodology were used to understand smokers’ perceptions of personal carbon monoxide monitors and accompanying smartphone apps [55]. Although some smokers were interested in using a personal monitor, others expressed concerns about testing in public (e.g., anticipated embarrassment) and about carrying around the device, which may be interpreted to suggest that the personal monitors are not (yet) perceived as acceptable to target users. In addition, focus groups were used to understand pre-use acceptability of a prototype smartphone app underpinned by machine learning algorithms to support individuals with binge eating disorder, with results indicating that participants were enthusiastic about the app and interested in testing it if it were to be developed [56].

Although important information may be gleaned from studies examining pre-use acceptability, we recommend following up with concurrent acceptability assessments, as users may not be able to predict how they will think and feel about the technology when testing it in their everyday lives.

## SUMMARY AND CONCLUSION

Here, we set out to discuss the ways in which acceptability may differ from related concepts, the utility of the concept of acceptability in digital health research and practice, the role of social and cultural norms and pragmatic means of measuring acceptability. We did not intend to offer solutions to these open questions but to initiate a debate within the digital health community. In addition, although we drew on a range of theoretical and empirical examples to support our arguments, this was not a systematic review of the available literature and our arguments are therefore unlikely to be exhaustive.

First, we argued that acceptability may usefully be considered an emergent property of a complex, adaptive system of interacting components, which in turn influences (and is influenced by) user engagement. Second, we argued that it is important to consider acceptability due to its ability to predict and explain outcomes of interest, including user engagement, intervention effectiveness and the scaling up of digital health interventions. Third, the types of digital health interventions that people find acceptable is deeply contextualized, ever changing and influenced by prevailing social and cultural norms. However, it is still useful to generate frameworks and approaches for improving acceptability, drawing on principles of user centered design. Finally, we argued that there is a lack of standard measures and thresholds against which to determine whether a digital health intervention is acceptable to users. However, brief star ratings and accompanying free-text responses, think aloud methodology, interviews and focus groups may provide pragmatic means of assessing people’s reactions to potential or available digital health interventions. We welcome further conceptual and empirical work to clarify the concept of acceptability within digital health research and practice.
